# Buying and selling human eggs: infertility providers’ ethical and other concerns regarding egg donor agencies

**DOI:** 10.1186/s12910-016-0151-z

**Published:** 2016-11-08

**Authors:** Robert Klitzman

**Affiliations:** Columbia University, 1051 Riverside Drive #15, NY 10032 New York, USA

**Keywords:** Ethics, Policy, Informed consent, Infertility, IVF, Oocyte donation, Oocytes, Assisted reproductive technology

## Abstract

**Background:**

Egg donor agencies are increasingly being used as part of IVF in the US, but are essentially unregulated, posing critical ethical and policy questions concerning how providers view and use them, and what the implications might be.

**Methods:**

Thirty-seven in-depth interviews of approximately 1 h were conducted – with 27 IVF providers and 10 patients.

**Results:**

Clinicians vary in their views and interactions concerning egg donor agencies, ranging widely in whether and how often they use agencies. Agencies may offer egg recipients increased choices, but raise ethical and other concerns regarding respect for donors as individuals (e.g., adequacy of informed consent), potential harms, justice (e.g., concerns about possible eugenics – by encouraging and facilitating selection and marketing of facts for offspring), and donors constituting a vulnerable group. The quality of agencies appears to vary considerably, from acceptable to problematic. Agencies’ medical and psychological screenings of donors can range, and be minimal. Not all agencies adequately track donors’ prior numbers of donations, or share the relevant records with clinics. Clinics may find that potential donors have genetic mutations and medical problems about which they were unaware. Yet agencies and clinics do not provide care for such donors, generating stress. Dissemination of donors’ personal data can potentially threaten confidentiality. Questions emerge of whether increased monitoring/oversight of agencies may be beneficial.

**Conclusions:**

These data, the first to examine providers’ views and interactions regarding egg donor agencies, suggest wide variations in quality and use of agencies, and have critical implications for practice, policy, education and research. Given the potential limitations of the current model of self-regulation of agencies, the present data suggest needs to consider stronger professional guidelines or possible governmental regulations to establish, require and enforce higher standards for agencies to follow, regarding advertising to potential donors and recipients, arranging for appropriate informed consent concerning risks and benefits involved, and for quality control. Appropriate informed consent should be obtained from potential egg donors, including the fact that they may learn about mutations or medical problems about which they were unaware, but for which they will not receive treatment as part of this process. Enhancing understanding among the public-at-large about what egg donation entails may also be helpful.

**Electronic supplementary material:**

The online version of this article (doi:10.1186/s12910-016-0151-z) contains supplementary material, which is available to authorized users.

## Background

Assisted reproductive technology (ART) has posed a range of critical ethical concerns, but perhaps none more dramatically and controversially, as buying and selling human eggs. The US is one of the only countries in the world that permits compensation for oocytes. All countries in the European Union ban such compensation (except for reimbursement of minimal basic expenses such as for time, inconvenience and transportation [1]. Consequently, hundreds of egg donor agencies have been established in the US, but remain essentially unregulated, posing ethical questions [2–5].

Approximately 10 % of the population in all countries are infertile and many women delay childbirth in order first to pursue careers, often leading to fertility problems. Single individuals, as well as gay and lesbian couples, are also now using IVF (in vitro fertilization) to have children. Approximately 10.5 % of ART treatments in the United States (US) use eggs from donors [6]. These issues are also of broader significance outside the US, since egg donors may be paid in other countries (e.g., India) [7], and many foreigners travel to the US for infertility treatment, and purchase eggs.

In the US, IVF clinics are essentially self-regulated, with guidelines published by the American Society for Reproductive Medicine (ASRM) addressing compensating, recruiting and communicating risks/benefits to egg donors [8]. ASRM states, for instance, that “it would be prudent to limit donors to those who are 21 or older and have the emotional maturity to make such decisions” [9]. In 2007, the organization recommended that donors do not undergo the procedure more than six times in their lifetime (Practice Committee of the ASRM and Practice Committee of the Society of Assisted Reproductive Technologies [SART] [10] – partly since donation carries medical risks, including 1.2 % risk of ovarian hyperstimulation syndrome [11], and questions remain as to whether fertility drugs that donors must take may increase risks of uterine or other cancers [12].

Compensation should not vary according to the planned use of the oocytes retrieved, the number or outcome of prior donation cycles, or the donor’s ethnic or other personal characteristics; and total payments to donors in excess of $5,000 require justification and sums above $10,000 are not appropriate.

But in 2013, Lindsey Kamakahi sued ASRM, alleging that these guidelines for payments constituted price fixing; and in 2016, ASRM settled the lawsuit [13–15], eliminating recommendations concerning caps on compensation or egg donation.

Yet while the amounts of compensation have been examined, other critical questions concerning how egg sellers interface and interact with ART providers have received far less attention. Agencies exist as third-party companies outside of professional medicine and are not regulated by state or the federal government [16] or subject to professional codes of conduct, and are even less influenced by a model of self-regulation. The only effort made to regulate agencies regarding these issues has consisted of ASRM allowing agencies to agree voluntarily to that organization’s guidelines in exchange for a listing on ASRM’s website as a professional endorsement. Yet this initiative has only partially led participating agencies to comply fully with these guidelines. Research suggests that most agencies do not follow these recommendations. Agencies advertise widely in college newspapers, and advertisements of prices for eggs in college newspapers increase with the average SAT scores of the school [5], raising concerns about eugenics-related searches for “the best genes.” Among a study of agency and clinic ads on Craigslist, 81 % of agency and 96 % of clinic ads were non-compliant with ARSM guidelines, including 85 % of those agencies and clinics that were SART-registered [17]. Most egg donor agencies fail to comply with ASRM’s guidelines that bar varying compensation based on a donor’s traits, with 58.8 % explicitly stating that they pay more for certain traits, and an additional 17.6 % stating that certain traits are “preferred” or “in demand.” The most commonly-mentioned compensated trait was prior donation success. While ASRM recommended not using donors under 21 years of age, 45.8 % of agency websites sought younger donors.

Though the American Medical Association guidelines require that health websites present risks alongside compensation, 98 % of the agency websites presented compensation amounts, but 74 % did not mention psychological and/or emotional risks, none mentioned possible cancer risks, 47.1 % failed to mention short-term risks, and 88.7 % did not mention possible risk to future fertility. Among websites of IVF clinics and egg donor agencies, 92.2 and 58.8 %, respectively, were SART or ASRM approved, but 39.7 % of these were still inconsistent with ASRM guidelines, with 25.6 % explicitly paying more for certain traits and 14.1 % stating that certain traits were “preferred” or “in demand”. Agencies were more likely than clinics to offer a range of fees rather than a fixed amount (78.4 % vs. 13.7 %), to specify “preferred” traits (76.5 % vs 21.6 %), and to fail to acknowledge a possible cancer risk (100 % vs. 92.2 %).

Agency websites often seek to influence potential donors to see donation as natural and fulfilling; and attempt to influence potential donors through both monetary and non-monetary benefits that may inappropriately focus donors and recipients on personal gain, rather than on relevant medical considerations. These companies portray egg donors in familiar, reassuring ways to assuage recipients’ fears about using reproductive material from strangers, including in donor profiles a range of donor personality characteristics, physical or artistic talents and intellectual abilities, passion and sense of purpose in life, and general temperament or demeanor, and promulgate misunderstandings, making emotional appeals that can distract recipients from appropriate assessments of risks and benefits, and raising issues related to commodification, commercialization over professionalism, and eugenics. Indeed, Ameling interviewed staff at two egg donation agencies, who described encouraging donors to present “properly feminine profiles” [18]. These messages may, however, inappropriately inject emotional cues into donors’ and recipients’ decisions.

Yet strikingly, these past findings do not appear to have altered professional or other guidelines or policies. In part, understanding and awareness of these issues may remain relatively limited. Hence, additional data and insights concerning these companies, that affect attitudes and practices of countless individuals, can potentially help enhance comprehension of, and attention to, these businesses among providers in various fields, patients, policymakers, and the public more broadly.

In particular, the past data raise crucial questions that have not yet been explored concerning how ART providers themselves in fact view and interact with agencies as ethical issues arise. Providers may play central roles as gatekeepers concerning egg recipients’ use of agency donors. Hence, clinicians’ perspectives and experiences concerning agencies are critical. But no studies have examined how ART clinicians themselves see and interface with these businesses. These domains are especially critical at the present time since the settlement of the Kamakahi case, eliminating caps on amounts of compensation, as a result of which many more women may now seek to sell their eggs, and to do so through agencies. Thus, as part of a study on views of ART providers and patients toward several key aspects of IVF, issues concerning egg donor agencies were examined as well.

## Methods

In brief, as summarized on Table 1, and described more fully elsewhere as well [19], 37 in-depth semi-structured telephone interviews of approximately 1 h each were conducted in the US with 27 ART providers – 17 physicians and 10 other providers (7 mental health providers, 2 nurses, and 1 other) – and 10 patients. One physician and three other providers were also themselves patients.

**Table 1:** Characteristics of Sample

	Male	Female	Total
Physicians	14	3	17
Physicians who are also patients	0	1	1
Type of Practice			
University affiliated	5	1	6
Private Practice	9	2	11
Other ART providers (e.g., mental health providers, nurses)	1	9	10
Other providers who are also patients	0	3	3
Patients	1	9	10
Total	16	21	37

Interviews (for sample questions, see Additional file 1) explored participants’ views and decisions regarding ethical issues concerning egg donation and other key aspects of ART, and were systematically analyzed to obtain detailed descriptions of these issues. Since the participants were from throughout the US, interviews were conducted by telephone. The Columbia University Department of Psychiatry Institutional Review Board approved use of an information sheet about the study that was sent to all interviewees, who then provided verbal consent, which was documented by the researcher.

Since no prior studies have been published examining IVF providers' attitudes and practices concerning egg donor agencies, qualitative methods were chosen because these can best elicit the full range and typologies of attitudes, interactions and practices involved, and can inform subsequent quantitative studies. Qualitative methods have been used successfully to reveal critical aspects of patient attitudes and practices concerning views of other topics related to IVF – e.g., patients’ decisions concerning disclosures of donor oocytes [20].

From a theoretical standpoint, Geertz has advocated studying aspects of individuals’ lives, decisions, and social situations not by imposing theoretical structures, but by trying to understand the individuals’ own experiences, drawing on their own words and perspectives to obtain a “thick description” [21]. The methods for the present study adapted elements from “Grounded Theory” [22] and were thus informed by techniques of “constant comparison,” with data from different contexts compared for similarities and differences, to see if they suggest hypotheses. This technique generates new analytic categories and questions, and checks them for reasonableness. These methods have been used in several other studies on key aspects of health behavior and doctor-patient relationships and communications in genetics and other areas [23–27]. During the ongoing process of interviewing, the Principal Investigator (PI) constantly considered the ways participants resemble or differ from each other, and the social, cultural, and medical contexts and factors that contribute to differences. Grounded Theory also involves both deductive and inductive thinking, building inductively from the data to an understanding of themes and patterns within the data, and deductively, drawing on frameworks from prior research and theories.

### Participants

Patients and providers were recruited through listservs, emails, and word-of-mouth. Providers were also recruited through national ASRM meetings (e.g., PGD and mental health provider interest group meetings). The PI approached these meeting attendees to ascertain whether they might be interested in participating in an interview study, and if so, the PI subsequently emailed them information about it. Most of those asked agreed to participate, and did so. A mental health listserv was also used, which is received by approximately 60 members (not all of whom are active), of whom 15 responded, and the first 8 respondents were then interviewed. Additional interviews were conducted as background, for informational purposes, with 8 physicians, 9 mental health providers and 14 patients; and informed, but were not included in the final formal data analysis. Interviews for the formal data analyses were conducted with each group until “saturation” was reached (i.e., “the point at which no new information or themes are observed in the data” [28]). Interviewees were from across the US. Providers described interactions with multiple patients they had treated, and colleagues; and patients often described interactions with multiple providers and other patients.

### Instruments

The semi-structured interview questionnaire was drafted drawing on prior literature, and explored patients’ and providers’ views, experiences and decisions concerning multiple aspects of ART, including use of egg donor agencies (See Appendix for sample questions).

### Data analysis

Transcriptions and initial analyses of interviews occurred during the period in which the interviews were being conducted, enhancing validity, and helped shape subsequent interviews. Once the full set of interviews was completed, subsequent analyses were conducted in two phases, primarily by trained research assistants (RAs) and the PI. In phase I, they independently examined a subset of interviews to assess factors that shaped participants’ experiences, identifying categories of recurrent themes and issues that were subsequently given codes. The PI and RAs read each interview, systematically coding blocks of text to assign “core” codes or categories (e.g., views of use of egg donor agencies, or interactions with them). While reading the interviews, a topic name (or code) was inserted beside each excerpt of the interview to indicate the themes being discussed. The PI and RAs then worked together to reconcile these independently developed coding schemes into a single scheme. Next, a coding manual was prepared, defining each code and examining areas of disagreement until reaching consensus. New themes that did not fit into the original coding framework were discussed, and modifications made in the manual when deemed appropriate.

In phase II of the analysis, the PI and RAs independently content-analyzed the data to identify the principal subcategories, and ranges of variation within each of the core codes. They reconciled the sub-themes identified by each coder into a single set of “secondary” codes and an elaborated set of core codes. These codes assess subcategories and other situational and social factors. Such subcategories included, for instance, views of egg donor agencies as satisfactory or as problematic; and the quality of screenings of donors by agencies.

Codes and sub-codes were then used in analysis of all of the interviews. To ensure coding reliability, the PI and an RA analyzed all interviews. Where necessary, multiple codes were used. Similarities and differences were assessed between participants, examining categories that emerged, ranges of variation within categories, and variables that may be involved. Areas of disagreement were examined through closer analysis until consensus was reached. Regularly for consistency and accuracy in ratings was checked regularly by comparing earlier and later coded excerpts.

Text from the interviews is presented below to allow readers to appreciate the richness of the data obtained.

## Results

**Fig. 1:**
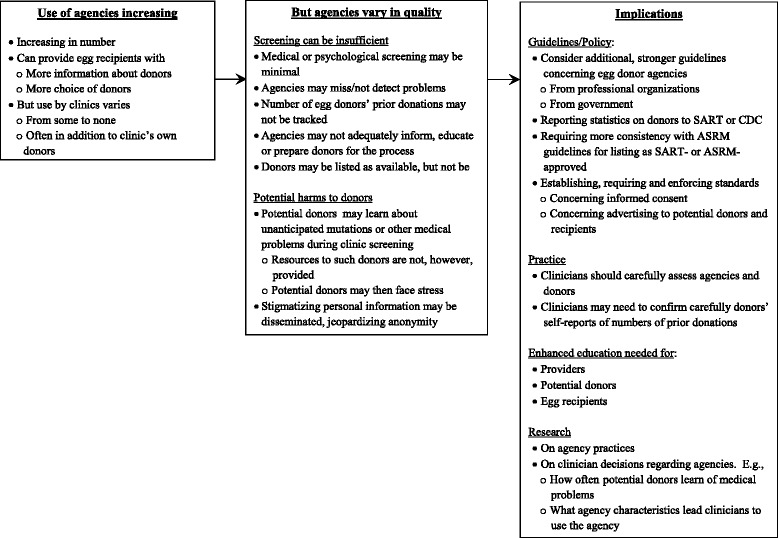
Issues concerning egg donor agencies

Overall, as outlined in Fig. 1 and described more fully below, egg donor agencies appear to be increasing and changing; but clinicians vary in how they view, and whether and how often they use, these companies – from rarely to often. Agencies pose critical ethical concerns regarding informed consent, potential harms, justice (related to possible eugenics), and donors constituting a vulnerable group. Providers perceived variations in the quality of agencies. Medical and psychological screenings of donors can range, and be minimal or lacking. Agencies may not adequately track how many times a woman has previously donated, and/or may list a donor as available when she is not. Clinics may find that a donor has medical issues about which she was unaware, causing her stress. The dissemination of personal data about donors can pose potential confidentiality problems. Questions arise of whether increased monitoring or oversight of agencies may be beneficial.

### Increasing use of agencies

As the demand for donor eggs has risen, agencies may be increasing, evolving, and affecting clinics’ practices. Due to agencies, patients often have more choices and information about donors than before. Providers have frequently altered their recruitment of donors and use of agencies and many clinics are increasingly working with these companies

Until recently, donors were anonymous. The clinics chose the donors. But market forces have changed things. The egg donor agencies are really taking over. [Other provider #5]

Clinical practice has changed, increasing the use of agencies, due not to altered regulations, but to market forces, particularly the growth of agencies, giving prospective parents more choices. Agencies have made more information about prospective donors available, potentially affecting clinics’ practices as well.

Clinics used to not show adult pictures of donors. But now, prospective parents can see photos, and a lot of patients want to. With these agencies, there are more patients than before. [Other provider #5]

Many clinics may thus use agencies in order to give patients more of a choice of potential donors – often through websites.

When patients wanted to do donor egg, some clinics would find the egg donor, and introduce that egg donor, on paper, to the patient, and the patient would have to either take her or leave her. A lot of people want more say about who their donor is. They may leave that clinic, and come to a program where they can go to a donor agency, look at tons of profiles, and pick someone who really seems right for them. [Other provider-patient #10]

Clinics vary in how much and in what ways they work with egg donor agencies. Many providers prefer to use only donors they know and have used before, rather than those from agencies. “Most reputable clinics choose their own donors.” [Other provider #5]

Nonetheless, partly because finding good egg donors can be hard, other clinics may both recruit donors on their own, and use those from agencies.

I’m a nurse and have run the donor egg program at my clinic for eight years. We recruit our own donors, and have agency donors, too. The most difficult issues are the recruitment of good donors, and the times when recipients don’t get pregnant. [Other provider #7]

The fact that agencies generally offer much more information about egg donors, often publicly online, can pose challenges though for oocyte recipients and the future children. Offspring created using purchased eggs might eventually want to find their biological mother using publicly available online data and information. More knowledge about donors can decrease anonymity, and create and shape expectations that children will receive these donors’ desired traits, raising expectations for the child.

Patients might come across the donor on the street. It’s complicated. And there’s a limit in choosing. If patients see the adult picture, it might distance them from the child as they see their child growing.” [Other provider #5]

Yet questions emerge of whether these choices, based on physical traits (e.g., height and blonde hair and blue eyes), and educational institutions (e.g., Ivy League) and attainment may at a certain point begin to raise concerns about possible eugenics.

### The quality of agencies varies

Agencies also range widely in quality, and some may be better than others – in how well they evaluate, screen and prepare potential donors. In part, as a result, providers differ in their views of agencies.

A few providers had good experiences with agencies with which they have worked, establishing on-going relationships.

I haven’t worked with that many agencies. I’ve liked the ones I know, that the clinic uses. Recently, we used an agency and it turned out that the egg donor didn’t produce good eggs that could be fertilized. We would never have predicted it. The agency said it’s only happened to them once. [Other provider-patient #10]

Yet other providers have also experienced this problem of donors not ultimately producing sufficient usable eggs, despite the agency having suggested otherwise, suggesting that this problem may be more widespread than this interviewee suspects..

Many clinicians had more mixed or negative views and experiences concerning agencies. Agencies may, for instance, list as donors women who have characteristics that appeal to potential recipients, but turn out to be unavailable.

Providers also often felt that egg donor agencies did not always adequately screen donors. Clinicians thus frequently conducted their own assessments of all potential donors, including those from agencies.

Several clinics evaluate donors very carefully – much more so than do agencies. We have a strict program for our donors. We test donors a lot. One agency said I reject donors that have donated elsewhere. We send our donors’ profiles to a geneticist, and do extensive psychological testing. We regulate it ourselves. [Other provider #7]

Clinics may thus screen far more vigorously than do agencies.

Agencies may also not fully inform, educate and prepare prospective donors regarding the process, and the risks involved. Financial profit may motivate certain agencies, lowering their standards regarding which donors they accept, and how well they assess these women.

A lot of these egg donors are not being vetted properly. It’s big money. With the egg donor agencies, anybody can sign up on-line. There’s minimal screening. People can lie. Donors come in, and are surprised at the depth of my questions and the amount of detail. And I’m only asking them standard questions! They’ll say they did a psychological test on-line, which is really unethical, because a psychological test is supposed to be monitored. They’ll say, “Well, I met with somebody.” Or: “I’ve met with someone in the hallway for 10 minute,” or “The medical doctor met with me and said ‘everything’s going to be fine.’” But the doctor didn’t check whether the donor understood what she would be undergoing. Then they met with another psychologist or a social worker, and it was much briefer. Or there was nobody. Or they went to the clinic, and signed up. Who does quality control? With egg donor agencies, any female of the right age could sign up on-line. Basically if somebody applies, the agency will send a packet for them to fill out. They may send them to be evaluated by someone, have them do an abbreviated on-line psychological test. It’s not hard to be put “on the books” as a potential donor. They get often flown to a different clinic, and by then, potential parents are invested in them. [Other provider #5]

Problems surface because agencies do not fully or adequately probe potential donors’ personal or family medical histories. Clinicians then have questions that they come to probe further.

I see things on evaluations from the agencies that lead me to question donors more. On one egg donor evaluation, everything looked great. The father had been dead for a number of years. “What did he die of?” “He was murdered.” Given that information, I would want to get a good idea about what happened. [Other provider-patient #10]

### Potential harms to donors

Practices of agencies could also potentially harm donors, who come either to learn about medical problems about which they were unaware, or they may face threats to confidentiality. Since agency screening may be minimal and/or superficial, some potential donors may suddenly learn for the first time, from a clinic, that they are in fact unsuitable, and/or have, or are at risk of, serious genetic problems. These potential donors then confront unexpected stress.

Most patients go by the donor’s looks or education – mostly looks – but I’ve turned down likeable, attractive donors, saying: “We’re here to rule out passing certain mutations to recipients. There are a number of reasons why I may not think it’s a good idea for you to proceed. This is not personal. It doesn’t mean I don’t like you, or that you’re not nice. But there are guidelines.” [Other provider #5]

Yet these donors had anticipated gaining financially, not learning about their own genetic risks or diseases. Agencies often do not inform donors about these potential pitfalls.

They get screened, and find out they have a genetic problem that’s going to affect their own future! And they’re not chosen as a donor, and don’t get paid! Nobody says, “Here, go to this nice mental health professional, and process how you just found out you’ve got this problem.” They should go talk to someone, but won’t. That’s a bum rap. Donors are looking for money, but can’t say that. They have to say they’re altruistic, or they won’t get chosen. But most are financially motivated. [Other provider #5]

Stigmatized, personal medical information about donors may also get spread, threatening their confidentiality.

We’ve seen confidentiality problems. By HIPAA [the Health Insurance Portability and Accountability Act], we can only give information we find about the donor to the donor, not the agency. But the agency then calls the donor, and asks her a lot of questions. She’s young and vulnerable and gives answers. The agency then gives out that medical information, which could be venereal disease testing, genetic testing, or drug testing, to the recipients. That is probably not illegal, because the agencies are not professionals – so they don’t have to behave according to professional guidelines or ethics. So they give information about one person to another. [Physician #14]

U.S. laws bar health care providers and institutions, but not egg donor agencies, from sharing personal information about potential donors with others – including clinicians, prospective parents and the public-at-large. Many agencies provide photos and extensive information about egg donors on websites, and/or send copies to prospective egg recipients and clinicians.

### Policy implications: overseeing agencies?

Concerns arose that agencies do not always adhere to, or help promote, key parts of current guidelines – for instance, not monitoring the maximum number of times a woman can sell her eggs.

Guidelines aren’t necessarily being followed. The guidelines say egg donors are only supposed to donate six times, but a lot of agencies don’t respect that, despite what it can do to women’s bodies. I’ve read on the Internet and seen books by donors who say they don’t tell agencies what they’ve been through. Nor do the agencies necessarily ask. Major medical centers ask. For-profit agencies may not. [Other provider-patient #9]

Many – but not all – providers may then exercise heightened caution regarding potential donors’ numbers of prior donations, probing for this information very thoroughly. Yet other clinicians may simply accept the agency’s report of the potential donor’s past history regarding donations and other relevant behaviors.

It’s difficult to be 100 % sure that donors from agencies keep track of the number of cycles. One donor told me she’s never donated before. But she knew too much about egg donation. She seemed to think that the injections were gonna be a piece of cake. I said, “Something’s not adding up here. What’s going on?” She then told me she “had a cycle done elsewhere,” and “wasn’t happy” with the way they treated her. [Other provider #7]

Consequently, this provider now regularly checks records of women who have donated elsewhere. But agencies do not always agree to give this crucial information.

I check records every time I can get them – 60 % of the time – because we use a lot of agency donors. When an agency donor has done a previous cycle, I’ve gotten the records in every case – except when the program won’t release them to me. [Other provider #7]

At times, questions arise of whether egg donor agencies should be overseen in some way, though doing so presents challenges, obstacles and questions.

I would find some way to put the brokers and agencies out of business. But you’d have to figure out how to do that. I would put this aspect of medical care into the hands of medical professionals, and out of the hands of business people! [Physician #14]

The fact that egg agencies are involved with medical procedures, but are not operated by medical professionals can thus create problems.

## Discussion

These data, the first to explore how ART providers view and interact with egg donor agencies, raise several critical ethical issues. Clinicians often use agencies in order to have more supply of eggs, and give patients more choices; but agencies appear to vary widely in quality – in how well they screen, inform and prepare donors for the process. These companies may not assess or record well how many times donors have previously provided oocytes; and may not agree to share the relevant records with clinics. Potential donors may end up learning about medical problems about which they were unaware; yet agencies and clinics generally do not provide care or resources for further assessment or treatment, and such potential donors may then confront unforeseen stresses. Moreover, dissemination of donors’ personal data can potentially threaten confidentiality.

This study builds on earlier research on studies of agency advertisements and staff [19, 29, 30], but offers for the first time data on provider perspectives and experiences, suggesting additional ethical concerns regarding many agencies – problems that have not been previously reported – e.g., regarding the quality of screenings and potential harms. These data suggest that not all agencies are deficient, but many appear to be so. While some clinicians may screen potential donors with added care, other providers may not do so as well. Agencies increase choices in numbers of egg donor traits, but may also foster both misunderstandings about genetics, and eugenic-like notions that may have harmful, longer-term effects. These data also pose concerns, post-Kamakahi, about undue inducement, with more women seeking to sell their eggs through agencies for increasing amounts of money, without fully considering the various potential risks involved. In particular, many agencies and clinics may not fully or appropriately inform, educate or vet these young women. Egg donation is more invasive than sperm donation, requiring medications and needles, and thus involves more risk (i.e., of Ovarian Hyperstimulation Syndrome [31]), heightening concerns.

These data are from the US, but have important implications for other countries. Though many European countries ban payment to egg donors beyond limited compensation for time and expenses [32], citizens of these and other countries may travel to the US to purchase eggs; and in upcoming years, agencies may well be established in additional countries across the globe that may regulate the egg market little, if at all.

These findings have critical implications for future practice, education, guidelines and research. Specifically, these findings suggest that further guidelines, oversight, or regulation may be beneficial. Though currently, agencies may register with ASRM, 41.2 % do not do so, and most of those that do so in fact violate ASRM guidelines [33, 34]. ASRM could potentially require that agencies register with the organization in order to work with its members; and more strongly emphasize needs to adhere fully to ASRM guidelines. Professional medical organizations could also more strongly recommend that agencies fully inform potential donors about the benefits and risks of donation – including the possibility that potential donors may learn about medical problems for which treatment will not be provided by ART clinics. It is crucial that egg donors provide appropriate informed consent, which necessitates that they fully understand the potential risks involved. Unfortunately, it appears that donors at many agencies may not always do so. Clinics themselves should also provide this information; but as Kahneman and Tversky [35] have described, an “anchoring heuristic” exists, whereby initial information that individuals receive “anchors” or establishes a mental framework that shapes how later information is weighed, molding subsequent decisions [36]. Hence, informing potential donors initially about all relevant risks and information is vital.

ASRM or other policymakers could also mandate that agencies adequately screen potential donors, specifying components of appropriate screening; carefully track the total number of times donors have contributed oocytes and provide this information to clinics, if requested; and only list as available those donors who are indeed available. Such recommendations could help ensure that the highest possible ethical standards are followed. Moreover, agencies could report key data to SART or Centers for Disease Control (CDC) annually, as ART clinics do – e.g., the number of women who donate each year, the number of times each woman has donated, and any complications that arise, to allow for enhanced transparency and help in monitoring and overseeing this growing industry. The CDC could require that all agencies provide such data.

Given the potential limitations of the current model of self-regulation, the present findings suggest needs to consider stronger professional guidelines or possible governmental regulations to establish, require and enforce higher standards for agencies to follow, regarding advertising to potential donors and recipients, arranging for appropriate informed consent concerning risks and benefits involved, and for quality control. Given that the system of self-regulation has had limited effectiveness, with current ASRM guidelines routinely ignored by many egg donor agencies, and that no legal redress appears to exist for failing to comply with the current guidelines, much stronger enforcement of guidelines or governmental regulations at least need to be considered as options. The federal government (through the CDC or Federal Drug Administration) or states (through departments of health) could require that all agencies register, report data, and become certified. State departments of education and professional licensing could require that egg donor agency owners and/or key employees become licensed, which could require formal legal agreements to adhere to the highest possible standards.

Providers should recognize the wide differences that exist in the quality of agencies; and should proceed with care. Education of patients and potential egg providers is also vital, to highlight needs for caution regarding the practices of many – though not all – agencies. Appropriate informed consent should be obtained from potential egg donors, including the fact that they may learn about mutations or medical problems about which they were unaware, but for which they will not receive treatment as part of this process. Enhancing understanding among the public-at-large about what egg donation involves may also be helpful.

These data suggest both needs for future research and a research agenda, to investigate further, using larger samples, how often and which clinics use agencies; which characteristics of agencies lead providers to use these companies; what kinds of screening agencies conduct of potential donors, and how it can be improved; how frequently physicians reject potential donors and why (e.g., how often potential donors are found to have serious mutations or other medical problems); and how well egg donors understand the processes and potential risks involved. These data also underscore, more broadly, how qualitative data can illuminate the “on the ground” experiences of providers and patients, revealing ethical challenges, complexities, and nuances they confront.

This study has several potential limitations. The sample size is sufficient for qualitative research designed to elucidate the issues and themes that emerge; however, future studies using larger samples are needed to analyze statistically several aspects of these issues (e.g., which clinics do vs. do not use agencies). Though it is potentially conceivable that providers with certain attitudes may have been more likely to participate for some reason, the interviewees here demonstrated a full range of attitudes. Nonetheless, future studies can explore these issues with larger samples. However, physicians and other clinicians are very busy, and increasingly difficult to recruit for surveys, as indicated by response rates of surveys of providers declining significantly over time [37, 38]. Recruiting larger numbers of them can pose challenges, no doubt accounting in part for the lack of any prior studies on these critical questions, and making the present data of added value. Arguably, these data also have a certain face validity, illuminating challenges that arise.

## Conclusions

These data, the first to examine providers’ views and interactions regarding egg donor agencies, suggest wide variations in quality and use of agencies, and have critical implications for practice, policy, education and research. Given the potential limitations of the current model of self-regulation of agencies, the present data suggest needs to consider stronger professional guidelines or possible governmental regulations to establish, require and enforce higher standards for agencies to follow, regarding advertising to potential donors and recipients, arranging for appropriate informed consent concerning risks and benefits involved, and for quality control. Appropriate informed consent should be obtained from potential egg donors, including the fact that they may learn about mutations or medical problems about which they were unaware, but for which they will not receive treatment as part of this process. Enhancing understanding among the public-at-large about what egg donation entails involves may also be helpful.

## Additional file

Additional file 1: Sample Questions for Providers. (DOCX 14 kb)

## Abbreviations

ART: Assisted reproductive technology
ASRM: American Society for Reproductive Medicine
CDC: Centers for Disease Control
FDA: Federal Drug Administration
HIPAA: The health insurance portability and accountability act
IVF: In vitro fertilization
PI: Principal Investigator
RA: Research assistant
SART: Society of assisted reproductive technologies
