# Supplementary material for: Buying and selling human eggs: infertility providers’ ethical and other concerns regarding egg donor agencies
Source: BMC Med Ethics. 2016 Nov 8;17:71. doi: 10.1186/s12910-016-0151-z (PMC5101668; doi:10.1186/s12910-016-0151-z)
Supplement: Additional file 1: — Sample Questions for Providers. (DOCX 14 kb) [file 12910_2016_151_MOESM1_ESM.docx]

Supplementary File 1: Appendix

**Appendix: Sample Questions for Providers**

- What challenges do you face in your work as an ART provider?
  - How do you address these challenges?
- Have you faced challenges concerning procuring oocytes from donors? If so, when? What was different about the situation? What did you do? How did you make these decisions?
- Have you faced challenges concerning egg donor agencies? If so, when? What was different about the situation? What did you do? How did you make these decisions?
- How do you view these issues?
- How have your patients viewed these issues?
- What additional thoughts do you have about these issues?
